# A Synergistic Effect of Lp(a) and GRACE Score on Cardiovascular Risk in Acute Coronary Syndrome Patients Undergoing Percutaneous Coronary Intervention: A Cohort Study From China

**DOI:** 10.3389/fcvm.2021.637366

**Published:** 2021-02-19

**Authors:** Chengping Hu, Jinxing Liu, Hongya Han, Yan Sun, Yujing Cheng, Yan Liu, Ang Gao, Yujie Zhou, Jianwei Zhang, Yingxin Zhao

**Affiliations:** Department of Cardiology, Beijing Institute of Heart Lung and Blood Vessel Disease, Beijing Anzhen Hospital, Capital Medical University, Beijing, China

**Keywords:** lipoprotein(a), acute coronary syndrome, percutaneous coronary intervention, GRACE score, prognosis

## Abstract

**Objectives:** Lipoprotein(a) [Lp(a)] has been thought as an independent risk factor for atherosclerotic cardiovascular disease (ASCVD). The Global Registry of Acute Coronary Events (GRACE) score is used to predict the risk of death or death/non-fatal myocardial infarction in patients with acute coronary syndromes (ACS). It suggests that there may be a synergism between Lp(a) and the GRACE risk score on predicting cardiovascular events. Accordingly, this study aimed to test the hypothesis that Lp(a)-related cardiovascular risk could be significantly modulated by the GRACE risk score in patients with ACS undergoing percutaneous coronary intervention (PCI).

**Methods:** Patients hospitalized with ACS undergoing PCI were enrolled and followed up for 18 months. The primary outcome was the composite of death, non-fatal myocardial infarction, non-fatal stroke, and unplanned repeat revascularization. A Cox proportional hazard regression model was used to determine the relationship between Lp(a) and cardiovascular events.

**Results:** A total of 6,309 patients were included (age: 60.1 ± 10.06 years, male: 75.2%, BMI: 26.2 ± 10.57 kg/m^2^). A total of 310 (4.9%) cardiovascular events occurred. When the overall population was stratified by a GRACE score of 91 or less vs. more than 91 and by tertiles of Lp(a), higher Lp(a) was significantly associated with cardiovascular events only when the GRACE score was <91(tertile 2 vs. tertile 1: HR 1.31, 95% CI: 0.86–1.98, *P* = 0.205; tertile 3 vs. tertile 1: HR 1.94, 95% CI: 1.32–2.84, *P* = 0.001; *P* = 0.002). However, no such significant correlation between cardiovascular events and Lp(a) emerged in the case of a GRACE score 91 or less, and there was a significant interaction for cardiovascular events between Lp(a) tertiles and dichotomized GRACE scores (*P* < 0.001).

**Conclusions:** In ACS patients undergoing PCI, there was a synergistic effect between the GRACE risk score and on-statins Lp(a) on predicting cardiovascular events. This finding could help us more accurately identify patients who would benefit most from Lp(a)-lowering treatment.

## Introduction

Despite effective low-density lipoprotein cholesterol (LDL-C)-lowering treatment, a significant residual risk remains. In IMPROVE-IT (Improved Reduction of Outcomes: Vytorin Efficacy International Trial), the rate of cardiovascular risk remained 32.7% even after LDL-C reached 54 mg/dl or less, suggesting that LDL-C-lowering treatment might not optimally reduce cardiovascular risk (1). Lipoprotein(a) [Lp(a)] has been considered an independent risk factor for atherosclerotic cardiovascular disease (ASCVD), and high Lp(a) remains a risk factor despite LDL-C being <70 mg/dl (2–4). The JUPITER trial (Justification for the Use of Statins in Prevention: An Intervention Trial Evaluating Rosuvastatin) enrolled 9612 patients and indicated that higher on-statin Lp(a) was associated with greater cardiovascular risk independent of low-density lipoprotein cholesterol (LDL-C) [adjusted hazard ratio (HR) 1.27; 95% confidence interval (CI), 1.01–1.59, *P* = 0.04] (2). Thus, Lp(a) was considered a risk factor and potential therapeutic target (5–7).

Evidence has suggested that Lp(a) could be reduced effectively by ~20–40% for proprotein convertase subtilisin/kexin type 9 (PCSK9) inhibitors, cholesteryl ester transfer protein inhibitors, and mipomersen and by 70–90% with antisense oligonucleotides (8). Despite recent progress, which patients are likely to benefit the most from a reduction in Lp(a) remains uncertain.

Lp(a) is believed to have pro-thrombotic properties, suggesting that there might be a synergistic effect of Lp(a) and systemic thrombotic risk on cardiovascular events. The Global Registry of Acute Coronary Events (GRACE) score has a very good discriminative ability for predicting death in patients with ACS regardless of ST-segment elevation myocardial infarction (STEMI), non-ST-segment elevation myocardial infarction (NSTEMI), or unstable angina (9). The National Institute for Health and Care Excellence (NICE) independently tested all of the risk scores (GRACE, TIMI, PURSUIT, PREDICT, EMMACE, SRI, AMIS, UA risk score) in 64,312 patients and demonstrated that the GRACE score performs significantly better than other risk scores with a c statistic of 0.825 (95% CI 0.82–0.83) (10). In addition, current guidelines also recommend the GRACE score to calculate patients' short-term and long-term risks of fatal or non-fatal cardiovascular events (11). Therefore, we employ the GRACE post-discharge risk score to assess the risk of cardiovascular events in this study.

Accordingly, we tested the hypothesis that Lp(a)-related cardiovascular risk could be significantly modulated by the GRACE score in patients with acute coronary syndromes (ACS) undergoing percutaneous coronary intervention (PCI), and it could better identify patients who would benefit most from Lp(a) lowering.

## Methods

### Study Design and Patients

From January 2018 to December 2018, ACS patients hospitalized for PCI were enrolled in this study. The key exclusion criteria were a body mass index (BMI) > 45 kg/m^2^, suspected familial hypertriglyceridemia (triglyceride ≥5.65 mmol/L), severe hepatic and renal insufficiency (eGFR <30 ml/min), left ventricular ejection fraction (LVEF) <30%, and use of fibrate and PCSK9 inhibitors which have great effects on Lp(a) and malignancy. Moreover, patients with incomplete key variables including GRACE score variables and Lp(a) were also excluded. The institutional review board of Beijing Anzhen Hospital, Capital Medical University, approved the study protocol. A waiver of informed consent was granted, and patients' personal information was concealed.

### Measurements

Patients fasted for at least 8 h before blood draw. Blood was drawn on the day of admission for fasting patients, and the next morning for non-fasting patients. Moreover, measurement was performed on the day of drawing blood. For patients with STEMI, blood samples were collected immediately on admission. Lipid profiles were measured on the same day of collection. Lipid parameters, glycosylated hemoglobin (HbA1c), and fasting plasma glucose (FPG) were quantified by clinical standard laboratory techniques. ELISA (Biocheck Laboratories, Toledo, OH, USA) was performed to detect Lp(a) and high-sensitivity C-reactive protein (hs-CRP) levels. In addition, the GRACE post-discharge score with eight variables was calculated to estimate the risk of cardiovascular events (12).

### Treatment and Procedures

Aspirin and clopidogrel or ticagrelor was given preoperatively and unfractionated heparin (70–100 IU/kg) intraoperatively. All of the patients took statins with or without ezetimibe unless serious complications occurred. Coronary intervention was performed using 6 or 7 F guiding catheters through a radial approach. Balloon pre-dilation was followed by second-generation drug-eluting stents. The type of stent, optical coherence tomography (OCT), intravascular ultrasound (IVUS), and fractional flow reserve (FFR) were left to the discretion of the interventionalists. All medications and operations were performed in compliance with current guidelines (13).

### Outcomes

Follow-up for 18 months was performed by telephone conversation, and the time to index event was used for analysis. The hospital records were also provided data for screening clinical events. The primary outcome for the current analysis was a composite of all-cause death, non-fatal myocardial infarction (MI), non-fatal stroke, or unplanned repeat revascularization. Death was defined as any death resulting from any cause. Incident stroke was defined as acute cerebral infarction according to the typical symptoms or imaging (14). Incident MI was defined on the basis of the fourth universal definition of myocardial infarction (15). Unplanned revascularization was defined as any unexpected PCI or surgical bypass after the index procedure on either target or non-target vessel (14, 16). Unstable angina was defined as acute chest pain with or without electrocardiographic abnormalities and normal cardiac enzymes (17). Dyslipidemia was defined as self-reported use of any lipid-lowering drug, fasting TG > 150 mg/dL, HDL-C <40 mg/dL, and/or LDL-C > 130 mg/dL. Diabetes was defined as taking hypoglycemic agents, a fasting plasma glucose of ≥7.0 mmol/L, non-fasting plasma glucose of ≥11.10 mmol/L, or self-reported disease (18). Hypertension was defined as use of anti-hypertensive drugs, systolic blood pressure ≥140 mm Hg, or diastolic blood pressure ≥90 mmHg (19).

### Statistical Analysis

The baseline characteristics are presented according to the median baseline GRACE score. Continuous variables are presented as the mean ± standard deviation (SD) or median (interquartile range) and were compared with the *t* test or the Mann–Whitney *U* test. Categorical variables are reported as numbers (percentage) and compared using the χ^2^ test (Fisher's exact test). Cox proportional hazard regression models were used to determine the relationship between cardiovascular events and Lp(a) with the backward stepwise method as a variable selection method, and the model was conducted by fully adjusting for variables, including age, sex, BMI, smoking status, hypertension, previous MI, previous stroke, SYNTAX score, number of stents, total length of stents, high sensitivity C-reactive protein (hs-CRP), HDL-C, LDL-C, and lipid-lowering medication use. The statistical interaction between Lp(a) and GRACE score was examined by incorporating multiplicative interaction terms in the same model. The Kaplan–Meier curves were plotted to illustrate the cumulative incidence of cardiovascular events over time, and they were compared by the log-rank test. Sensitivity analyses were performed in patients with or without diabetes. Moreover, we provide the comparisons of baseline characteristics between participants who were included in the final analyses or not to test whether the lost data were random. Statistical analyses were performed using SPSS software, version 24.0 (IBM Corp., Armonk, NY, USA). Statistical significance was considered as *P* < 0.05 (two-tailed).

## Results

A total of 9,285 patients met the inclusion criteria, with 2,976 excluded due to loss to follow-up (*n* = 698) or the exclusion criteria (*n* = 2,278). Thus, a total of 6,309 patients were included in the final analysis. The patient flowchart is shown in Supplementary Figure 1. Supplementary Table 1 illustrates the comparison of baseline characteristics between ineligible and eligible patients. Though statistically significant, differences in SBP, current smoking status, hypertension, and lipid parameters were not clinically relevant. In addition, the table shows no significant differences in sex, BMI, hs-CRP, or GRACE risk score.

### Baseline Characteristics

The baseline characteristics are displayed in Table 1. The patients were 75.2% male, with a mean (SD) age of 60.1(10.06) years old, and a mean (SD) BMI of 26.2 (10.57) kg/m^2^. The rates of diabetes and dyslipidemia were 44.4% (2,803) and 74.7% (4,710), respectively. A total of 5,488 (87%) patients were presented with unstable angina. A total of 6,155 (97.6%) patients received aspirin, and 6,200 (98.3%) took a statin with/without ezetimibe (20.1%). Left main artery lesions were observed in 16.8%, multivessel lesions in 58.9%, CTO lesion in 16.7%, and lesions > 20 mm in 61% of patients. When we evaluated baseline characteristics according to median GRACE score, patients with a GRACE score >91 vs. 91 or less had higher BMI; had more cardiovascular risk factors including current smoking, hypertension, diabetes, low-density lipoprotein cholesterol levels, TC, and TG; and were less likely to be receiving statins. The rates of female and AMI were higher in the high-GRACE group. Besides, patients in the high-GRACE group were likely to have a higher SYNTAX score and there was no statistically significant difference for other angiographic parameters.

**Table 1:** Baseline characteristics of patients according to GRACE score.

	**Total**	**Grace score** **≤91**	**Grace score** **>91**	***P-*value**
*N* (%)	6,309	3,365 (53.3)	2,944 (46.7)	–
Age, y	60.1 ± 10.06	54.1 ± 8.16	67 ± 7.23	<0.001
Male, n (%)	4,747 (75.2)	2,703 (80.3)	2,044 (69.4)	<0.001
BMI, kg/m^2^	26.2 ± 10.57	26.7 ± 12.38	25.7 ± 7.88	<0.001
Heart rate, bpm	72.3 ± 11.5	71.4 ± 11.22	73.3 ± 11.73	<0.001
SBP, mmHg	1,28.2 ± 21.12	129.3 ± 19.02	126.9 ± 23.23	<0.001
**MEDICAL HISTORY AND RISK FACTORS**, ***n*** **(%)**				
Current smoker	2,286 (36.2)	1,394 (41.4)	892 (30.3)	<0.001
Hypertension	4,097 (64.9)	2138 (63.5)	1959 (66.5)	0.013
Diabetes	2,803 (44.4)	1392 (41.4)	1,411 (47.9)	<0.001
Dyslipidemia	4,710 (74.7)	2,489 (74)	2,221 (75.4)	0.179
Previous MI	780 (12.4)	272 (8.1)	508 (17.3)	<0.001
Previous stroke	293 (4.6)	96 (2.9)	197 (6.7)	<0.001
Previous PCI	1,542 (24.4)	771 (22.9)	771 (26.2)	0.003
Previous CABG	160 (2.5)	60 (1.8)	100 (3.4)	<0.001
**LABORATORY TESTS**				
Cr, μmol/L	77.6 ± 49.75	74.7 ± 44.57	81 ± 54.91	<0.001
eGFR, ml/min/1.73 m^2^	123.6 ± 36.2	130.9 ± 38.9	115.4 ± 30.84	<0.001
FPG, mmol/L	7 ± 2.58	6.9 ± 2.55	7.2 ± 2.59	<0.001
HbA1C, %	6.6 ± 1.38	6.5 ± 1.36	6.7 ± 1.38	<0.001
TC, mmol/L	4.1 ± 1.07	4.1 ± 1.09	4.1 ± 1.04	0.004
TG, mmol/L	1.4 (1.0–2.0)	1.5 (1.1–2.2)	1.0 (1.3–1.9)	<0.001
HDL-C, mmol/L	1.1 ± 0.25	1.1 ± 0.24	1.1 ± 0.26	0.001
LDL-C, mmol/L	2.4 ± 0.89	2.4 ± 0.91	2.4 ± 0.86	0.005
LP(a), mg/dL	13.0 (5.0–31.0)	12.0 (5.0–31.0)	13.0 (6.0–31.8)	0.859
hs-CRP, mg/L	0.5 (0.2–1.6)	0.4 (0.1–1.2)	0.6 (0.2–2.2)	0.01
TNI, μg/L	0.9 ± 5.34	0.8 ± 5.54	1 ± 5.11	0.284
LVEF,%	61.2 ± 7.76	62.4 ± 6.62	59.7 ± 8.73	<0.001
GRACE score	90.4 ± 20.96	75 ± 12.53	108 ± 13.47	<0.001
**ACS type**, ***n*** **(%)**				
Unstable angina	5,488 (87)	3,067 (91.1)	2,421 (82.2)	<0.001
AMI	821 (13)	298 (8.9)	523 (17.8)	<0.001
Killip class at admission, *n* (%)				0.708
I	513 (8.1)	189 (5.6)	324 (11.0)	
II–III	308 (4.9)	109 (3.2)	199 (6.8)	
**MEDICATION AT DISCHARGE**, ***n*** **(%)**				
Aspirin	6,155 (97.6)	3,288 (97.7)	2,867 (97.4)	0.401
Clopidogrel	4,433 (70.3)	2,271 (67.5)	2,162 (73.4)	<0.001
Ticagrelor	2,013 (31.9)	1,164 (34.6)	849 (28.8)	<0.001
ACEI/ARB	2,743 (43.5)	1,458 (43.3)	1,285 (43.6)	0.798
β-Blocker	4,069 (64.5)	2,152 (64)	1,917 (65.1)	0.336
Statin	6,200 (98.3)	3,321 (98.7)	2,879 (97.8)	0.006
Ezetimibe	1268 (20.1)	686 (20.4)	582 (19.8)	0.542
Any antidiabetic agents	2,245 (35.6)	1,112 (33)	1,133 (38.5)	<0.001
**ANGIOGRAPHIC CORONARY ANATOMY**, ***n*** **(%)**				
Any left main disease	1,059 (16.8)	549 (16.3)	510 (17.3)	0.285
Multivessel disease	3,715 (58.9)	2,003 (59.5)	1,712 (58.2)	0.269
Others	2,384 (37.8)	1,256 (37.3)	1,128 (38.3)	0.419
CTO	1,052 (16.7)	558 (16.6)	494 (16.8)	0.834
Lesions > 20 mm	3848 (61)	2,015 (59.9)	1,833 (62.3)	0.053
SYNTAX score	14 ± 7.48	13.5 ± 7.37	14.6 ± 7.58	<0.001
**Treated vessel**, ***n*** **(%)**				
LM	645 (10.2)	341 (10.1)	304 (10.3)	0.801
LAD	3,267 (51.8)	1,752 (52.1)	1,515 (51.5)	0.632
LCX	1,813 (28.7)	948 (28.2)	865 (29.4)	0.29
RCA	2,550 (40.4)	1,358 (40.4)	1,192 (40.5)	0.915
DCB	395 (6.3)	201 (6)	194 (6.6)	0.313
FFR	52 (0.8)	32 (1)	20 (0.7)	0.234
IVUS	150 (2.4)	71 (2.1)	79 (2.7)	0.136
OCT	124 (2)	79 (2.3)	45 (1.5)	0.019
Number of stents	1.7 ± 0.82	1.7 ± 0.83	1.7 ± 0.82	0.419
Total length of stents, mm	40.1 ± 24.48	39.7 ± 24.37	40.6 ± 24.61	0.4

*Values are shown as mean ± SD, median (interquartile range), or n (%). BMI, body mass index; SBP, systolic blood pressure; MI, myocardial infarction; PCI, percutaneous coronary intervention; CABG, Coronary Artery Bypass Grafting; Cr, creatinine; eGFR, estimated glomerular filtration rate; FPG, fasting plasma glucose; HbA1C, glycosylated hemoglobin; TC, total cholesterol; HDL-C, high-density lipoprotein-cholesterol; LDL-C, low-density lipoprotein-cholesterol; GRACE, Global Registry of Acute Coronary Events; Lp(a), lipoprotein(a); hs-CRP, high-sensitivity C-reactive protein; LVEF, left ventricular ejection fraction; AMI, acute myocardial infarction; ACEI, angiotensin-converting enzyme inhibitor; ARB, angiotensin II receptor blocker; CTO, chronic total occlusion; LM, left-main artery; LAD, left anterior descending artery; LCX, left circumflex artery; RCA, right coronary artery; DCB, drug-coated balloon; FFR, fractional flow reserve; IVUS, intravascular ultrasound; OCT, optical coherence tomography*.

### Relationships of Cardiovascular Events With Lp(a)

The fully adjusted multivariable relationships between cardiovascular events and Lp(a) levels stratified according to GRACE score are shown in Table 2. A total of 310 (4.9%) incident cardiovascular events occurred during 18 months of follow-up. Of the overall population, higher GRACE scores (≥91 vs. <91) were associated with an increased risk of cardiovascular events (HR: 1.50, 95% CI: 1.19–1.89, *P* < 0.001). However, no significant cardiovascular risk was associated with Lp(a) levels according to Lp(a) tertiles (tertile 2 vs. tertile 1: HR 1.11, 95% CI: 0.83–1.49, *P* = 0.472; tertile 3 vs. tertile 1: HR 1.33, 95% CI: 0.99–1.76, *P* = 0.051). Nevertheless, when the overall population was stratified by GRACE score of 91 or less vs. >91 and tertiles of Lp(a) levels, a higher Lp(a) level was significantly associated with cardiovascular events only when the GRACE score was >91 (tertile 2 vs. tertile 1: HR 1.31, 95% CI: 0.86–1.98, *P* = 0.205; tertile 3 vs. tertile 1: HR 1.94, 95% CI: 1.32–2.84, *P* = 0.001; *P* = 0.002). Moreover, no such significant correlation between cardiovascular events and Lp(a) emerged in the case of GRACE score of 91 or less. We also found that there was a significant interaction for cardiovascular events between Lp(a) tertiles and dichotomized GRACE score (*P* < 0.001).

**Table 2:** Risk of cardiovascular events according to lipoprotein(a) and GRACE score.

	**No. (%)**	**HR (95% CI)**	*P-*value^*^
LP(a) tertiles			
T1 (≤ 7 mg/dL)	92 (4.2)	1 (reference)	0.138
T2 (7–23 mg/dL)	100 (4.9)	1.11 (0.83–1.49)	0.472
T3 (>23 mg/dL)	118 (5.8)	1.33 (0.99–1.76)	0.051
**GRACE score**			
≤ 91	130 (3.9)	1 (reference)	-
>91	180 (6.1)	1.50 (1.19–1.89)	0.001
GRACE score ≤ 91			
LP(a) tertiles			
T1 (≤ 7 mg/dL)	49 (4)	1 (reference)	0.643
T2 (7–22 mg/dL)	44 (4.1)	0.96 (0.64–1.45)	0.85
T3 (>22 mg/dL)	37 (3.5)	0.82 (0.53–1.27)	0.366
GRACE score>91			
LP(a) tertiles			
T1 (≤ 7 mg/dL)	43 (4.4)	1 (reference)	0.002
T2 (7–24 mg/dL)	56 (5.7)	1.31 (0.86–1.98)	0.205
T3 (>24 mg/dL)	81 (8.2)	1.94 (1.32–2.84)	0.001

*GRACE, Global Registry of Acute Coronary Events; Lp(a), lipoprotein(a)*.

* *Adjusted for age, sex, BMI, smoking status, hypertension, previous MI, previous stroke, SYNTAX score, number of stents, total length of stents, hs-CRP, HDL-C, LDL-C, lipid-lowering medication use. P value for interaction <0.001*.

**Figure 1:**
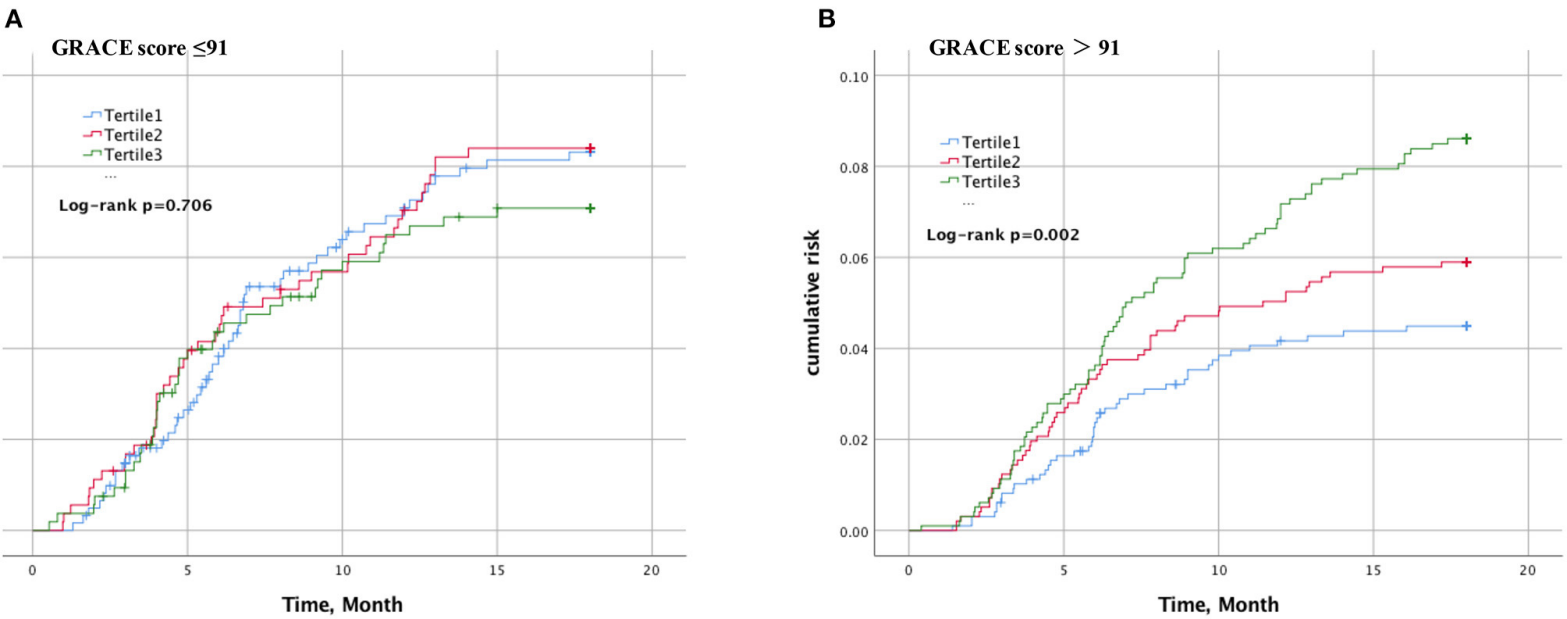
Kaplan–Meier curve of cardiovascular events according to lipoprotein(a) and GRACE score. The cumulative incidence of cardiovascular events over time stratified by Lp(a) tertiles in the case of GRACE score 91 or less **(A)** or >91 **(B)**.

The cumulative incidence of cardiovascular events over time stratified by Lp(a) tertiles in the case of GRACE score 91 or less (Figure 1A) or >91 (Figure 1B) during an 18-months period is shown. In patients with GRACE score >91, increasing Lp(a) was associated with a greater cumulative incidence of cardiovascular events over time (*P* < 0.002), whereas patients with GRACE score of 91 or less were not (*P* = 0.706).

Sensitivity analyses are described in Table 3 by presenting the associations between cardiovascular events and Lp(a) levels stratified according to GRACE scores in patients with or without diabetes. Similar to the overall population, higher Lp(a) levels in patients with diabetes were significantly associated with cardiovascular events only when the GRACE score was >92 (tertile 2 vs. tertile 1: HR 1.30, 95% CI: 0.73–2.31, *P* = 0.368; tertile 3 vs. tertile 1: HR 1.93, 95% CI: 1.14–3.25, *P* = 0.014; *P* = 0.039), but not when GRACE scores were 92 or less. However, no such relationship was found in patients without diabetes.

**Table 3:** Risk of cardiovascular events according to lipoprotein(a) and GRACE score in patients with or without diabetes.

	**Diabetes**		**Non-diabetes**	
	**HR (95% CI)**	*P-*value^*^	**HR (95% CI)**	*P-*value^*^
GRACE score ≤ median				
LP(a) tertiles				
T1	1 (reference)	0.308	1 (reference)	0.875
T2	0.70 (0.39–1.27)	0.244	1.16 (0.59–2.27)	0.675
T3	1.12 (0.65–1.92)	0.68	1.18 (0.60–2.34)	0.633
GRACE score > median				
LP(a) tertiles				
T1	1 (reference)	0.039	1 (reference)	0.151
T2	1.30 (0.73–2.31)	0.368	1.24 (0.71–2.18)	0.45
T3	1.93 (1.14–3.25)	0.014	1.67 (0.98–2.84)	0.059

*GRACE, Global Registry of Acute Coronary Events; Lp(a), lipoprotein(a)*.

* *Adjusted for age, sex, BMI, smoking status, hypertension, previous MI, previous stroke, SYNTAX score, number of stents, total length of stents, hs-CRP, HDL-C, LDL-C, lipid-lowering medication use*.

## Discussion

### Main Findings

Our findings demonstrated for the first time that there was a synergistic effect between GRACE score and on-statins Lp(a) levels on cardiovascular risk for ACS patients undergoing PCI. Lp(a)-mediated cardiovascular risk only appears in case of greater risk of cardiovascular events (GRACE score > 91), but no such relationship exists in case of GRACE scores of 91 or less.

Studies have shown that Lp(a)-lowering treatments might only be effective at high Lp(a) levels (20). However, the question is what the threshold is of Lp(a) for patients with a high cardiovascular risk. A meta-analysis including 7 randomized controlled trials (RCT) and 29 069 statin-treated patients found that patients with Lp(a) >50 mg/dL were associated with a 40% higher cardiovascular risk (21). Further, Lp(a) levels <50 mg/dl are recommended by the American Heart Association/American College of Cardiology cholesterol guidelines as optimal (5). However, as noted by the Copenhagen data (22) and randomized trials (2, 3), patients with 25–50 mg/dl Lp(a) still carry a high risk of cardiovascular events, which was ignored by the recommendation. Actually, Lp(a) levels <30 mg/dl might be deemed optimal and the risk could be almost negligible (8). For example, Erqou et al. (23) suggested that ASCVD risk starts to accrue from a Lp(a) level of 25–30 mg/dL. The present analysis suggests that a significant cardiovascular risk appears with an Lp(a) level of 24 mg/dL or more in the case of a GRACE score of 91 or more, providing additional evidence. In addition, patients with Lp(a) levels higher than 25–30 mg/dl accounted for 30% of the global population, ~2 billion people with atherogenic properties 8).

Prior evidence in patients without previous CVD from epidemiological studies (23), Mendelian randomization studies (24), and genome-wide association studies (25, 26) have conclusively shown that Lp(a) is closely related to cardiovascular risk. A study included a total of 63,746 coronary artery disease (CAD) cases and 130,681 controls, indicating that the most potent genetic association with CAD was the LPA locus, which was more potent than LDL-, PCSK9-, and 9p21-related variants (26). Epidemiological studies (24, 27, 28), genome-wide association studies, and Mendelian randomization studies (25, 29) have shown that patients with high Lp(a) were likely to have a greater risk of death, myocardial infarction, and stroke and the associations were causal. A meta-analysis included 11 studies for a total of 18,978 subjects with established CAD and found that Lp(a) was significantly associated with cardiovascular risk (30). A pre-specified analysis of the placebo-controlled ODYSSEY Outcomes trial in 18,924 patients with recent ACS indicated that Lp(a) predicted the risk of MACE after recent ACS and suggested that Lp(a) was an independent predictor of cardiovascular risk and should be a treatment target for patients with ACS (31). A *post hoc* analysis of the FOURIER trial (Further Cardiovascular Outcomes Research with PCSK9 Inhibition in Subjects With Elevated Risk) suggested that an increased risk of venous thromboembolism (VTE) was significantly related to the increased Lp (a) but not LDL-C levels (32). Our findings further indicated that the relationship between Lp(a) and cardiovascular events could be affected by the GRACE score. Actually, a previous study demonstrated that the relationship was also mediated by hsCRP levels, and the association only existed in patients with hsCRP levels ≥2 mg/L but not in those with hsCRP <2 mg/L (33).

However, the mechanisms responsible for the association between Lp(a) and cardiovascular risk remain uncertain. Lp(a) directly promotes the formation of atherosclerotic plaques as the same as LDL cholesterol because of the cholesterol component of Lp(a). Moreover, the structural homology of apo(a) with plasminogen can lead to pro-thrombosis/anti-fibrinolysis effects by interfering with endogenous fibrinolysis (22). In addition, apo(a) carries 85% of the pro-inflammatory oxidized phospholipids, which could damage the intima of coronary arteries and facilitate the rupture of plaques (34).

### Clinical Implication

Based on our findings, these data suggest that patients with Lp(a)-mediated cardiovascular risk could be further identified by selecting patients with greater risk of cardiovascular events. Thus, it could allow us to more accurately identify people who might benefit the most from LP(a)-lowering treatment.

### Limitations

This study had several limitations. First, although we adjusted for a wide range of confounders, residual confounding factors cannot be excluded in this cohort study. Second, the Lp(a) assay measured Lp(a) mass was less than ideal. Third, Lp(a) levels in this study were much lower than other studies in western countries. So the results might not be generalizable to other ethnic groups.

Fourth, it should also be noted that the proportion of patients with unstable angina in this study was much higher than in previous studies on ACS. Finally, the study was exploratory and the optimal cutoff value of the GRACE score and Lp(a) for identifying patients with high cardiovascular risk must be validated in RCTs.

## Conclusions

In patients undergoing PCI for ACS, there was a synergistic effect on cardiovascular risk between GRACE score and on-statins Lp(a) levels. This finding could help us to more accurately identify patients who would benefit most from Lp(a)-lowering treatments. However, the findings should be repeated in more randomized, controlled trials.

## Data Availability Statement

The original contributions presented in the study are included in the article/Supplementary Material, further inquiries can be directed to the corresponding author/s.

## Ethics Statement

The studies involving human participants were reviewed and approved by the institutional review board of Beijing Anzhen Hospital. The ethics committee waived the requirement of written informed consent for participation.

## Author Contributions

YZ, JZ, and CH contributed to the conception and design of this study. CH wrote this article. All authors made contribution to collect, analyze data, read, and approved the final manuscript.

## Conflict of Interest

The authors declare that the research was conducted in the absence of any commercial or financial relationships that could be construed as a potential conflict of interest.
