# Supplementary material for: A Synergistic Effect of Lp(a) and GRACE Score on Cardiovascular Risk in Acute Coronary Syndrome Patients Undergoing Percutaneous Coronary Intervention: A Cohort Study From China
Source: Front Cardiovasc Med. 2021 Feb 19;8:637366. doi: 10.3389/fcvm.2021.637366 (PMC7933013; doi:10.3389/fcvm.2021.637366)
Supplement: Supplementary file 1 [file Data_Sheet_1.docx]

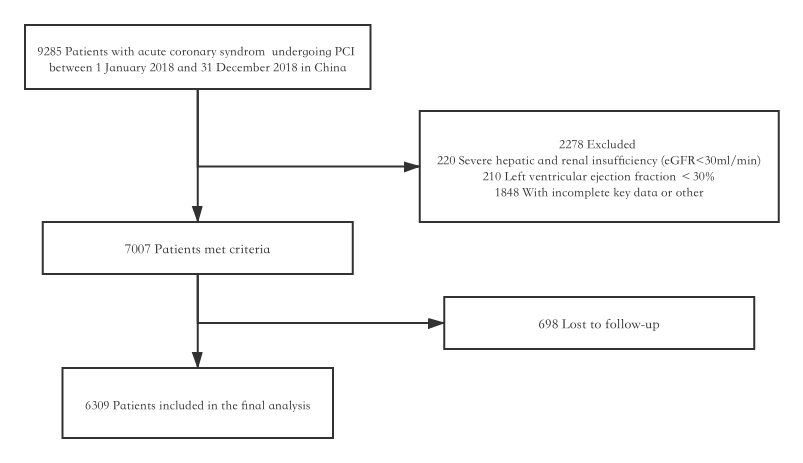


Supplementary Figure 1 flowchart

Supplementary Table 1 Baseline characteristics of the study participants who were eligible and unavailable for analysis

|  | Lost participants | Eligible participants | *P value* |
| --- | --- | --- | --- |
| N | 2976 | 6309 | - |
| Age, y | 59.4±10.01 | 60.1±10.06 | 0.001 |
| Male,n (%) | 2249(75.6) | 4747(75.2) | 0.731 |
| BMI,kg/m2 | 26.3±5.23 | 26.2±10.57 | 0.929 |
| SBP, mmHg | 129.3±20.29 | 128.2±21.12 | 0.022 |
| Medical history and risk factors, n (%) | |  |  |
| Current smoker | 1211(40.7) | 2286(36.2) | ＜0.001 |
| Hypertension | 2007(67.4) | 4097(64.9) | 0.018 |
| Diabetes | 1271(42.7) | 2803(44.4) | 0.119 |
| Dyslipidaemia | 2421(81.4) | 4710(74.7) | ＜0.001 |
| FPG,mmol/L | 7.1±2.67 | 7±2.58 | 0.674 |
| HbA1C,% | 6.6±1.42 | 6.6±1.38 | 0.588 |
| TC | 4.2±1.06 | 4.1±1.07 | ＜0.001 |
| TG | 1.8±1.48 | 1.7±1.25 | ＜0.001 |
| HDL-C | 1.1±0.26 | 1.1±0.25 | 0.311 |
| LDL-C | 2.5±0.87 | 2.4±0.89 | 0.003 |
| hs-CRP | 23.3±38.48 | 19.5±38.76 | 0.166 |
| GRACE score | 89.8±21.83 | 90.4±20.96 | 0.247 |

Values are mean± SD, median (interquartile range), or n (%). BMI body mass index, SBP systolic blood pressure, FPG fasting plasma glucose, HbA1C Glycosylated hemoglobin, TC total cholesterol, HDL-C high-density lipoprotein-cholesterol, LDL-C low-density lipoprotein-cholesterol, GRACE Global Registry of Acute Coronary Events; hs-CRP high-sensitivity C-reactive protein
